# PPIE in a technical research study: Using public involvement to refine the concept and understanding and move towards a multidimensional concept of disability

**DOI:** 10.1111/hex.14072

**Published:** 2024-05-15

**Authors:** William Lammons, Lucky Nobility, Sarah Markham, Eirini‐Christina Saloniki

**Affiliations:** ^1^ National Institute of Health and Care Research Applied Research Collaboration (NIHR ARC), North Thames London UK; ^2^ Department of Primary Care and Population Health, Institute of Epidemiology and Health Care University College London London UK; ^3^ Department of Biostatistics and Health Informatics King's College London London UK

**Keywords:** disability, focus groups, mapping, public involvement, surveys

## Abstract

**Background:**

Disability is often an essentialised and oversimplified concept. We propose refining this while incorporating the multidimensional nature of disability by increasing the use of existing survey questions and their corresponding data to enrich, broaden and inform understandings of disability.

**Methods:**

We combined patient and public involvement and engagement (PPIE) with focus groups and concept mapping to collaboratively map disability survey questions into conceptual models of disability with six members of the public with lived experiences of disability.

**Results:**

Using reflexive thematic analysis, we identified three qualitative themes and eight subthemes through a series of four PPIE activities: (1) understanding concepts of disability based on individual experience, subthemes: 1.1—preference for the biopsychosocial model, 1.2—‘Reviewing’ instead of mapping survey questions and 1.3—comparing questions to real life; (2) consistency between understanding needs and implementing adjustments, subthemes: 2.1—connecting preparation and operation, 2.2—inclusivity and adjustments in activities and 2.3—feedback for improving activities and (3) real‐world applications—targeted awareness raising, subthemes: 3.1—who, where, what and how to share activity findings and results, 3.2—sharing with human resource and equality, diversity and inclusion professionals.

**Conclusion:**

Members of the public who collaborated in these activities felt empowered, engaged and supported throughout this study. This approach offers a model for other researchers to cede power to the public over the research aspects typically reserved for researchers.

**Patient or Public Contribution:**

We involved members of the public with lived experience throughout this study—co‐design, co‐facilitation, collaboratively mapping the disability or disability‐related survey questions into conceptual models of disability, evaluation of the activities, co‐analysis and co‐authorship.

## 1 INTRODUCTION

### 1.1 Measuring disability

Measuring disability in the whole world is chiefly accomplished by empirical studies and their use of survey data, composed of individual questions asking respondents on specific attributes of their disability.^1^ However, this approach has been critiqued and problematised for essentialising and oversimplifying a multidimensional phenomenon.^2^, ^3^, ^4^ Meanwhile, individual survey questions related to disability can be difficult to identify and understand, and crucially less accessible for disabled people and their advocacy groups, due to different formats and lack of concentration in one site.

To reduce this complexity while acknowledging and incorporating the multidimensional nature of disability, increasing the use of existing survey questions and their corresponding data could enrich and broaden understandings of disability worldwide. Many survey questions addressing disability remain largely unused. Coincidentally, many of these questions have been designed with certain underlying conceptual models (i.e., groups of terms and definitions used to define and describe disability), yet this is scarcely made explicit. Identifying relevant disability or disability‐related questions followed by ‘mapping’ (i.e., categorising survey questions into conceptual models of disability) can be one way towards increasing uptick in using survey questions on disability, and therefore, improve understanding of the concept of disability.

In addition to mapping survey questions to conceptual models of disability, we endeavoured to conduct this activity with members of the public with lived experience of disability to add layers of validity and relevance to the connections drawn between underlying conceptual understandings of disability that survey questions convey.

### 1.2 Patient and public involvement

Mapping survey questions into conceptual models in the context of disability is an activity typically reserved for researchers and can prove difficult for members of the public to engage in, as they are less familiar with the relevant technical processes and concepts. We held a series of patient and public involvement and engagement (PPIE) activities with people of different lived experiences of disability to collaboratively render a technical research activity into a public one. PPIE offers a focus and methodology to make research a collaborative effort that considers and incorporates the public's perspectives and needs.^5^, ^6^, ^7^, ^8^ The activities we held included a combination of focus groups, concept mapping and evaluation.

PPIE employs a combination of qualitative research and project management to support collaborations centred around social interactions between researchers and members of the public.^9^, ^10^ It allows patients and stakeholders to formulate and co‐conduct research likely to affect them including, leading focus groups, shaping research protocols, reviewing results and designing dissemination plans.^6^, ^7^, ^8^, ^9^

Based on guidelines from the National Institute for Health and Care Research (NIHR), the research arm of the UK's National Health Service (NHS), the primary funder of health and social care research in the United Kingdom, and the entity who formalised and created guidelines for PPIE, we held a series of PPIE activities designed to familiarise these individuals with key concepts of mapping survey questions to conceptual models of disability, then cede decision‐making to them to make them partners in this technical activity.^10^, ^11^, ^12^, ^13^ This rendered them full collaborators in the research activities, a process that required a balance between bringing these concepts ‘down’ to the public's level through lay language and question‐answer opportunities and bringing these individuals ‘up’ to more technical discussions on which survey questions most closely fit with which models.^14^ Throughout this paper, we frequently refer to these members of the public who were involved in research activities as ‘public collaborators’ to emphasise their partnership in conducting this research with researchers.

### 1.3 Aims

The study's aims were to (a) identify all UK disability or disability‐related questions from the largest repository for secondary survey data, and (b) conduct a set of PPIE consultations to gain some understanding of public perceptions of theoretical models of disability in survey questions (via a mapping activity). This paper focuses on the public involvement methodologies, perspectives and analytical findings that emerged from the mapping activity.

## 2 MATERIALS AND METHODS

### 2.1 Co‐facilitation

Two public co‐authors, L. N. and S. M., acted as co‐facilitators for the activities, based on their lived experiences of disability and their collaboration via two different PPIE Panels, the Research Advisory Panel (RAP) and the Virtual Document Review Panel (VDRP).^15^ The RAP and VDRP support research for the NIHR Applied Research Collaboration (ARC) North Thames, an interdisciplinary research collaboration in the North Thames region (North London, Northeast London, East London, South Hertfordshire, South Bedfordshire, and Mid‐ and South‐Essex), which focuses on producing applied research for healthcare practice relevant to local communities.^16^

The co‐facilitators' experiences of disability allowed us to address ‘power differentials’ between those participating in the mapping activity and those leading it.^10^, ^17^ They were involved in every part of the study following the receipt of the grant funding—recruitment of participants, design of activities, explaining key concepts, reviewing discussions and critiquing the results. This helped address and support ‘differing priorities’ that come with varied lived experiences and incorporate them into the study.^17^

### 2.2 Recruitment

We publicised advertisements for the study activities through PPIE platforms (People in Research^18^) and relationships with disability charities (Scope and Shaping Our Lives^19^, ^20^).

We aimed to recruit people representing a variety of disabilities from across the North Thames region. Twenty‐two people expressed interest in the study via email, and we used a multistep process to evaluate and select session attendees: (a) individual meeting— W. L. and E.‐C. S. met individually with 10 of those who reached out via email and were based in North Thames to discuss the study in an informal interview format. The remaining 12 were based outside North Thames. Of these 10, 1 of these was not selected, at which point, we decided to invite an additional 4 individuals based outside North Thames with compelling interests and lived experience connecting to the project for individual meetings for a total of 13; (b) candidate selection meeting—all authors met to discuss candidate preferences and selected 7 participants. We chose a pool of candidates that reflected a variety of disabilities (so as not to prioritise one disability over others), the North Thames region, willingness to engage with complex ideas and tasks around conceptual models of disability, and diversity of ethnicities, ages and other identities. Two applicants based outside North Thames were allowed to act as public collaborators because of their lived experiences, history of PPIE work and connection to the study. Of the seven confirmed for participation, six attended. One cancelled for personal reasons.

### 2.3 Activity structure and design

We held a series of four focus groups with concept mapping elements in this study that combined qualitative methods and public involvement. This combination and ordering of activities helped create a progression of thought, collaboration and productivity. It allowed us to familiarise and prepare public collaborators in key concepts, then cede power and decision‐making to them, while also learning how their personal experiences impacted their choices and studying their experiences of the activities to improve for the future. These activities followed the following format: (1) Explanation, (2) Mapping, (3) Reflection and (4) Evaluation and feedback. We illustrate this structure in greater detail in Table 1.

**Table 1:** Activities structure.

Order	Phase name	Duration	Method base	Explanation	Documentation
1	Explanation (preparation phase)	2 h	Preparing public collaborators by giving them skills necessary to perform research tasks^11^, ^12^, ^13^	This was the only ‘preparation’ phase in which we prepped collaborators for the later concept mapping activities. In this activity, we explained the conceptual models of disability to the participants and helped them become familiar with core concepts around conceptual models of disability and surveys. No data, reactions or feedback were collected in this phase. PowerPoint materials were reviewed with the ARC North Thames' Virtual Document Review Panel and co‐facilitators, then circulated with attendees before the activity.	No recordings, note taking
2	Mapping (experimental phase)	2 h	Combination of focus groups, decision making, and concept mapping^9^, ^21^, ^22^	In this activity, we recapped aspects of the previous session, then held the actual concept mapping. Collaboratively map survey questions to conceptual models.	Recordings and note taking
3	Reflection (experimental phase)	1.5 h	Focus groups^21^	In this activity, we provided additional opportunities to discuss the previous mapping exercise. We discussed and reflected with collaborators on how and why they mapped the survey questions to the models as they did, and how the findings can be disseminated.	Recordings and note taking
4	Evaluation and feedback (evaluation phase)	1 h	Focus groups, evaluation^21^, ^22^, ^23^, ^24^, ^25^	The concluding activity to the project. Discussion for participants to evaluate and critique the activities as an overall PPIE exercise. We intentionally guided all discussion away from the conceptual models, themselves and instead focused on their impressions of the overall process and how we could improve this for the future.	Note taking

Abbreviations: ARC, Applied Research Collaboration; PPIE, patient and public involvement and engagement.

The first activity, Explanation, focused on prepping the public collaborators by explaining and familiarising them with essential parts of conceptual models of disability. We conceive of the three conceptual models, the social, medical, and biopsychosocial models through the lens of the International Classification of Functioning (ICF), Disability and Health of the World Health Organisation (WHO).^24^ Their descriptions, definitions and examples, as we explained them in activities, appear in Table 2.

**Table 2:** Conceptual models of disability.

Name	Description	Examples
Medical model	Disability is a consequence of a physical and mental restriction. It is the individual's responsibility to handle and manage the disability. It focuses on clinical intervention, or ‘medical treatment or procedure’, and rehabilitation. It ignores the influence of society.	Cannot work full time Legs do not work Can't climb stairs Has special needs Needs medication
Social model	Disability is something a person experiences. Barriers to participation are socially constructed through the physical environment (e.g., stairs), social attitudes (how people behave and think) or institutional practices (e.g., government offices, universities, companies). The emphasis is on promoting social change, such as improving physical accessibility or attitudes toward inclusion. It ignores the impact of impairment.	Physical and emotional barrier Attitudinal barrier Institutional and organisational barrier Information and communication barrier
Biopsychosocial model	Disability is broadly defined, collectively referring to impairments, activity limitations and participation restrictions Disability is caused by physical or biological problems that need to be treated by experts + society needs to find ways to include disabled people in different activities (social, economic, political) by supporting them and providing them equal opportunities Disability reflects human functioning as the interaction between an individual's health condition and the influence of environmental, contextual and/or personal factors. Roughly speaking, this combines the medical and social models.	None given during activities

For the experimental aspect of the project, in which we mapped the survey questions to conceptual models of disability, we used two activities, Activity 2, Mapping, and Activity 3, Reflection. Activity 2, Mapping, focused on connecting groups of survey questions to ‘conceptual models’ of disability. A full list of the survey question groups that public collaborators mapped is available in Table 3. One author compiled these survey questions through a three‐step systematic search and compilation process from the UK Data Service data repository.^26^ This included: (a) clarifying search terms to find relevant surveys, (b) sifting questionnaires from relevant surveys to discover relevant questions around disability and (c) extracting and clustering relevant questions into clear sets to be used in PPIE activities with public collaborators. Search terms included: [impair*], [disab*] and [long‐term ill* OR condition* OR disab* OR impair*] and covered a timeframe of 2010–2023 within the United Kingdom. To account for variation between questions as observed across the different surveys, alternative wordings for some question groups were included (bold font in Table 3).

**Table 3:** Survey question groups for Activity 2, Mapping.

*Set A* (*consider questions as a single group*)	*Set A* (*alternative wording*)
A1. Do you have any long‐standing physical or mental impairment, illness or disability? By ‘long‐standing’ I mean anything that has affected you over a period of at least 12 months or that is likely to affect you over a period of at least 12 months.	A1. Do you have any long‐standing illness, disability or **infirmity** – by ‘long‐standing’ I mean anything that has troubled you over a period **of time** or that is likely to affect you over a period **of time**?
A2. Does this/Do these health problem(s) or disability(ies) mean that you have substantial difficulties with any of these areas of your life?	A2. Does this illness or disability (Do any of these illnesses or disabilities) **limit your activities in any way**?
*Set B* (*consider questions as a single group*)	*Set B* (*alternative wording*)
B1. Do you have any physical or mental health conditions or illnesses lasting or expected to last for 12 months or more?	B1. Do you have any illness **or disability** that you expect will last for at least 12 months?
B2. Does your condition or illness/do any of your conditions or illnesses reduce your ability to carry‐out day‐to‐day activities?	B2. Does this condition limit your ability to carry‐out **normal** day‐to‐day activities **in any way**? **[Please consider whether you are affected while receiving any treatment, taking medication, or using any devices, such as hearing aid.]**.
*Set C* (*consider questions as a single group*)	*Set C* (*alternative wording*)
C1. Do you have any health problems or disabilities that you expect will last for more than a year?	C1. Could I just check, do you have any **long‐standing illness, health problem or disability that limits your daily activities or the kind of work that you can do**?
C2. Does this health problem affect the kind of paid work that you might do?	C2. Do you have any health problem that **limits the kind or amount of paid work you could do, should you want to**?
C3. Does this health problem affect the amount of paid work that you might do?	No alternative wording for this question
*Set D* (*consider separately*)	*No alternative wording in this section*.
D1. Can I just check, do you consider yourself disabled?	
D2. Are you/they registered as a disabled person (or as visually impaired) with the local council/social services?	
D3. May I check, is there anyone living with you who is sick, disabled or elderly whom you look after or give special help to, other than in a professional capacity (e.g., a sick or disabled (or elderly) relative/husband/wife/child/friend/parent, etc.)?	

In Activity 3, Reflection, we held a focus group discussion in which we reviewed, clarified and elaborated on the mapping decisions that the collaborators made in Activity 2. Finally, Activity 4, Evaluation and Feedback, also used a focus group method, but focused on understanding collaborators' experiences of the activities, ideas for applying findings and feedback on how to improve on these activities for the future. Focus groups especially allowed collaborators' thoughts and experiences to emerge from the discussion, chiefly through open‐ended inductive questions and creating a social space around the creation and formalisation of collaborators' ideas.^27^, ^28^ A complete list of discussion questions can be found in Supporting Information S1: Material 1.

We chose to combine the focus group method with an evaluation of the PPIE activities to understand the ‘quality’ of the PPIE at the conclusion, especially considering if these activities surrounding a more technical research activity still ‘worked for’ public collaborators, and to help plan for future directions of this study and similar others.^25^ We opted not to use a conventional evaluation model as put forth by Gibson et al.,^23^ preferring an open‐ended inductive organic discussion, as we felt that this would ease the cognitive burden on collaborators after sharing various types of new information with them throughout the process.

### 2.4 Co‐producing activities with co‐facilitators

At the early planning stages of the process, we sought to ‘co‐produce’ the delivery, structure and design of these activities with our two co‐facilitators. Albert et al. have illustrated the slipperiness of the terminology around ‘co‐production’, therefore,^29^ we follow their and Smith et al.'s recommendation of clarity around reporting our approaches and conceptualisations of co‐production.^30^

Co‐production on this study specifically refers to our act of sharing power around decision making regarding the activities' structure, delivery and design, the co‐analysis of the results and findings, the authoring of this paper and the application of its suggestions.^29^ We have aimed throughout the process to make our co‐facilitators true partners in the design, delivery, analysis and dissemination of these activities.

Specific suggestions incorporated into the activities, based on co‐facilitators' suggestions included (a) multiple questions and answers opportunities to support understanding the material throughout presentations, (b) multiple comfort breaks and (c) refrain from recording in the first session to create a welcoming environment and ease people into the activities. During the sessions, co‐facilitators took notes, but felt led to act as participants because of the intertwining their facilitation roles with lived experiences and interest in the subject matter. Their contributions to the discussion particularly helped with facilitation, as they often steered discussion back on‐topic towards the intended mapping activity or focus of the session.

Reasonable adjustments and accommodations (e.g., materials format, special software, comfort breaks, etc.) were discussed individually with each participant before the first session to best support inclusivity and accessibility throughout the study. We held all meetings via Zoom, as digital sessions allowed for broader participation across the country and greater accessibility for those whose participation might be affected by disabilities. This included contributing nonverbally via chat features, which were read aloud, and deactivating video for any reason. We held sessions in a group setting, but offered one‐to‐one options to support participants, though no one preferred this latter format. Not every attendee was present in every session, and each session had at least four attendees.

As mentioned, based on co‐facilitator input, we chose not to record first session to allow participants to become familiar with the activities' processes and us as facilitators, or the fourth session to provide a cognitive break allowing for easier reflection, while we did record Activity 2, Mapping, and Activity 3, Reflection, because of their strong focus on discussion.

### 2.5 Analysis

We conducted a thematic analysis of data from all activities. This included recording transcriptions and notes taken by facilitators and co‐facilitators. Three coders, W. L., L. N. and S. M., collaboratively conducted a ‘reflexive thematic analysis,’ in which they reviewed the data and periodically shared qualitative in vivo codes and ideas, allowing codes to organically emerge from engagement with the data.^31^ Coders used a combination of NVivo 12,^32^ and highlighting and comment functions in Microsoft Word. Codes emerged from authors' interactions with session transcripts, but they were not fixed. As Braun and Clarke describe, our codes changed throughout the analysis process, that is, several expanded, contracted, were renamed and collapsed into others.^31^

After all coders had reviewed and coded all data, they discussed and reviewed the thoughts, findings and understandings that emerged from their codes. They then compiled and organised these together in consensus as a first draft of salient narrative ‘themes’, prompting recursive updates to codes and understandings of data.^31^ Coders referred back to new codes and emerging thematic structure while articulating and refining the themes.^31^

In line with reflexive thematic analysis, we built on Braun and Clarke's call for pragmatism and flexibility in analysis of qualitative data. we argue that our data and analytic themes are validated by ‘information power’, as opposed to ‘thematic saturation’, based on the robustness of collaborators' contributions, the variety of their identities and experiences, the unique challenges of the activities and the compelling insights the activities provided.^31^ Finally, we used the Guidance for Reporting Involvement of Patients and the Public, Version 2, checklist^33^ (Supporting Information S1: Material 2) and the Consolidated Criteria for Reporting Qualitative Research^34^ (Supporting Information S1: Material 3).

## 3 RESULTS

The four activities included six participants (three men, three women) with lived experience of disability from across England (Greater London, Manchester and Yorkshire). This group is not meant to be representative of the general population, but rather provides a varied collection of lived experiences of disability within England. All disabilities represented by public collaborators are listed in Table 4. Some had multiple disabilities.

**Table 4:** Disabilities of public collaborators.

Mobility or physical ○ Kienbock's disease ○ Ablations and open‐heart surgery ○ Automobile accident injuries ○ Fibromyalgia ○ ‘Spinal condition’ ○ Endometriosis ○ Amputation Neurodiversity ○ Autism spectrum disorder (ASD) ○ Attention deficit hyperactivity disorder (ADHD) Mental health ○ Anxiety ○ Bipolar disorder Sensory impairment ○ Noise sensitivity ○ Glaucoma ○ Myopia

Analysis of data from these activities revealed three qualitative themes: (1) understanding concepts of disability based on individual experience, (2) consistency between understanding needs and implementing adjustments and (3) real‐world applications—targeted awareness raising. We elaborate on these and their eight subthemes below.

### 3.1 Theme 1: Understanding concepts of disability based on individual experience

In mapping the survey questions to the conceptual models of disability, collaborators leaned heavily on their own conceptualisations of disability, based on their own lived experiences, including conceiving of disability as a holistic or all‐inclusive concept, reviewing the survey questions by means other than the conceptual models and analysing questions in terms of their personal lived experiences.

#### 3.1.1 Subtheme 1.1—Preference for the biopsychosocial model

Overall, activity participants voiced support and preference for the biopsychosocial model, frequently describing disability as a holistic concept in the process, indivisible into discrete aspects like physical, social and mental. They often described the biopsychosocial as a way of linking the different aspects of disability.

…[In response to Question Set A, I prefer] the biopsychosocial [model], because you cannot have one … just on medical without including the social aspects as well … they are inextricably linked. (Collaborator 1)

I think that the biopsychosocial model not only looks into the condition, but also what society is bringing in, and [how] our society is going to support that condition. (Collaborator 3)

[In response to Question Set D] … it has got a bit of medical [model] but more of social [model], so I think I would go for the third one, bio[psychosocial]. (Collaborator 4)

They often described the social and medical models as being too singular or falling short in conceptualising disability and described the medical model as ‘impersonal’, ‘narrow’ or ‘treating me like a number’:

The social one ignores the impact of impairment, and the medical one … puts …the responsibility … on the individual … I would say that the bio[psychosocial] one combines the two. (Collaborator 2)

Question set A treats me almost like a number question. Question set B actually personalizes it … not just a national insurance number or a hospital number, or anything else like that. It is more inclusive to my way of thinking. (Collaborator 1)

#### 3.1.2 Subtheme 1.2—‘Reviewing’ instead of mapping survey questions

In addition to connecting the question groups to their own conceptions of disability, public collaborators used various techniques to break the questions down and make them more relatable to themselves. At times, this included parsing the question groups into individual questions and differentiating them from one another, instead of mapping them as a single group. Some participants also categorised the questions as ‘qualitative’ or ‘quantitative,’ even though this did not factor into the conceptual models' criteria. Other collaborators provided review‐like feedback of the questions, as if to provide a different draft or version of them, such as calling questions ‘tricky’ or ‘difficult’ to answer as a survey respondent:

‘Do you have auditory problems, or do you have other problems?’ You know, there are so many different disabilities that one could list there. (Collaborator 1)

#### 3.1.3 Subtheme 1.3—Comparing questions to real‐life

Overall, collaborators imagined the real‐life context in which they might encounter such a question:

[In response to Question set C] … if this were a questionnaire coming from, say, a public body such as an entitlements application … then that might help clarify the situation somewhat… (Collaborator 1)

One string of conversation focused on comparing the survey questions to public funding forms:

[In response to Question Set B] … these questions are very much like the PIP [Person Independence Payment] forms … asking people these questions could be triggering, and I don't use that word lightly … I think wording this again is very important … [the PIP form questions] are horrendous … demeaning, demoralizing and … stressful, and everybody dreads [them] … these questions are the sort of questions that you're asked [on those forms]. (Collaborator 2)

In other cases, public collaborators answered the questions as if in a real‐life circumstance to try and make sense of them:

[From Question Set D], ‘Are you registered as a disabled person with your local council?’ Yes, they are the ones who have issued me with a blue badge. (Collaborator 1)

### 3.2 Theme 2: Consistency between understanding needs and implementing adjustments

In the reflective and evaluative activities, activities 3 and 4, collaborators described the facilitators as having delivered on supporting participants' access needs, while acknowledging opportunities for improvements and additional means of support.

#### 3.2.1 Subtheme 2.1—Connecting preparation and operation

Several participants described feelings of consistency between the preparation discussions for the activities and their enactment:

[I] like that you, E.‐C.S., promised the slides a week before [the meeting] and then kept to that. (Co‐facilitator 1)

You wanted to get our opinion [on the questions and the conceptual models] so that we are sure of our options … your explanation helped me to understand the [conceptual] model… (Collaborator 3)

Other participants praised specific methods taken by the facilitators as supporting inclusivity, including taking breaks, pacing of the slides and general support:

I liked the provision of breaks. Lots of meetings just say, ‘Oh we are just gonna push through’. It really helps with my concentration… (Co‐facilitator 1)

#### 3.2.2 Subtheme 2.2—Inclusivity and adjustments in activities

Some collaborators attributed the inclusivity in the sessions and the consistency between the facilitators' understanding needs and implementing adjustments to building trust and empathy, anyielding benefits including learning, growth, and the chance to apply these in other PPIE activities:

[Benefits for me included] listening to how different people learn and share. It has been really interesting to see how people come together, how they disagree. It has been particularly rich within these sessions. It has been incredibly invaluable. (Co‐facilitator 2)

For a lot of us, we would never really have thought about the models of disability. I have learned a lot that I will take away and use in my other PPI groups about learning, questions, accessibility, PPI needs. [I] found it very different – you put yourself in our shoes. I liked the way you paced slides. (Collaborator 5)

#### 3.2.3 Subtheme 2.3—Feedback for improving activities

There was also feedback on how to improve activities, including more interactive sessions, for example, voting buttons or Mentimeter‐like digital platforms that allow listeners to post answers to questions during a presentation,^35^ a glossary, gradually introducing terms, holding the last session face‐to‐face and circulating the survey questions before the activities.

The co‐facilitators debated the merits of larger versus smaller groups for these activities:

If you are discussing things that are hard, difficult, worrisome, it will be harder in bigger groups, especially if you're trying to grapple with something. (Co‐facilitator 2)

I have a different opinion. If you have a larger group, it might go more slowly as more people would mean more questions. The fewer sessions you have, the more people will attend more of them. (Co‐facilitator 1)

Some reflected on the collaboration process and provided feedback around further support:

If you have done PPI before, you want to have a bit more knowledge about the terms being used. For somebody that is new, it can be a bit overwhelming. It depends where you are on your PPI journey. Ask people about what they would prefer or need to prepare for before the sessions. (Co‐facilitator 2)

### 3.3 Theme 3: Real‐world applications—Targeted awareness raising

Collaborators recommended that these activities' findings should be used to raise awareness broadly in organisations and entities connected to disability and disabled people. They advocated for considering these activities and their findings as a knowledge resource that can help others to understand concepts of disability and consider how to think about disability.

#### 3.3.1 Subtheme 3.1—Who, where, what and how to share activity findings and results

Some collaborators supported sharing results and learnings through more logical connections, including disability‐related advocacy groups, volunteer and charity organisations and policy entities, like AbilityNet. Other collaborators clarified though, that in sharing information and learnings with organisations, there is a need to understand what they would want and need:

…I have a fear we are living in an information age, and far too often we are being overloaded with information, which is why I suggested ask the relevant questions [to the charities and disabled people on their needs], but do not overload us with information. (Collaborator 1)

Collaborators gave examples of helpful information, with some explanations as to why:

Research methods would give me a sense of security in the findings. (Collaborator 4)

Showing how you searched the [survey] questions and the use of terms. Breaking it down into the different regions [in the UK] will be useful. (Collaborator 5)

…the research questions [for this study] and how you attempted to answer them. (Collaborator 1)

Include representation of diversity of who was asked [these questions]. (Collaborator 5)

In addition to recommended content, some recommended specific formats, including creative, practical, and engaging media, such as animations, podcasts, videos, slideshows and information sheets.

#### 3.3.2 Subtheme 3.2—Sharing with human resources (HR) and equity, diversity and inclusion (EDI) professionals

Other collaborators expanded the question of with whom this information should be shared to include practical areas such as professionals, departments, and initiatives around HR, quality improvement and EDI, including British Heart Foundation, Diabetes UK and Chartered Institute of Personnel and Development:

I think disability charities, they'll have some awareness [of these models and concepts around disability], but … the people who you should prioritize are the people who aren't aware, like HR departments in organizations, EDI teams, etc. … understanding disability within workplaces is quite a big thing now, so when you apply for a job, you fill in an EDI survey … in the past, I've had some really poorly worded questions to try and understand, ‘Are you disabled?’ … anyone who works in HR, I think it would be a good thing to help them understand the different ways of trying to understand who is disabled in your workforce. (Co‐facilitator 1)

## 4 DISCUSSION

### 4.1 Main findings

We conducted four PPIE activities around mapping groups of disability‐related survey questions to conceptual models of disability with six members of the public with lived experience of disability and two co‐facilitators, also with lived experience of disability. We identified three themes and eight subthemes through a series of four PPIE activities: Theme 1—understanding concepts of disability based on individual experience, subthemes: 1.1– preference for the biopsychosocial model, 1.2—‘Reviewing’ instead of mapping survey questions, 1.3—comparing questions to real life; Theme 2—consistency between understanding needs and implementing adjustments, subthemes: 2.1– connecting preparation and operation, 2.2—inclusivity and adjustments in activities, 2.3—feedback for improving activities and Theme 3—real‐world applications—targeted awareness raising, subthemes: 3.1—who, where, what and how to share activity findings and results, 3.2—sharing with HR and EDI Professionals.

Disability is amongst the foundational inspiring concepts for PPIE and PPIE collaborators' calls for improving care, including those excluded from the research process and designing care pathways through collaborative research.^36^, ^37^ PPIE continues to be conducted robustly around disability, though this often occurs around a condition, experience or type of disability, such as dementia,^38^ learning disability^39^ or communication disability.^40^, ^41^ Our findings cut across differing experiences of, at times, multiple disabilities to examine and improve understanding of the disability concept itself. Like recent work, our findings advocate for co‐produced or collaboratively conducted research on disability with those who have and continue to experience disability.^40^, ^42^ Several publications show that doing so successfully and inclusively requires flexibility in public involvement methods to support the access needs of disabled members of the public and the pursuits of the research.^39^, ^40^, ^43^

We offer concept mapping combined with co‐design, co‐production and public involvement as one such flexible combination of methods that researchers focusing on disability can employ to support public involvement. Other researchers have recently taken similar approaches combining concept mapping with collaborative methods in creating a patient engagement evaluation toolkit,^44^ online group learning^45^ and health promotion at gyms or health clubs.^46^

This work contributes to a robust and growing literature on patient and public involvement by offering an example of how to share power and decision making in an activity rarely conducted with involvement of public collaborators with lived experience. To do so required the combination of co‐production, PPIE evaluation, focus groups, qualitative analysis, conceptual models and survey question mapping. Compelling work has been conducted on PPIE and evaluation,^25^ co‐production^47^ and co‐design,^48^ but this study builds on these examples by shifting members of the public more into a research‐focused activity and prioritising their perspectives and contributions.

This study reveals how complicated it can be to disconnect lived experience from research activities for some members of the public and the need for them to analyse things in their own ways, even while using a clear structure and methodology. As illustrated in reflexive anthropological studies, one's own subjectivity, identities, and experiences often reveal greater truths and understandings about social phenomenon than positivist methodologies.^49^, ^50^, ^51^ Even though we aimed to get people to exclusively map the research questions to the conceptual models, they took their own identities and lived experiences reflexively and openly into account in the process. Specifically, to assign the survey questions to a model, collaborators needed to go through a process of personal sense‐making. This meant reflecting on their own lived experiences and relating the questions to themselves. Essentially, they were mapping the question by thinking about how it applied to them in the real world.

Collaborators held a compelling interest in the biopsychosocial model. In unique and differing ways, they found this model to be more dynamic and holistic, meaning that it supported more complex ways of thinking about disability through various layers, some of which included medical testing and treatment, accessibility, qualifying for benefits, and visibility. They found this model relatable and more reflective of their own conceptions of their disabilities and lived experiences. Perhaps, this is why it was difficult for them to ‘separate’ their lived experiences from the mapping activity ‐ what they know and think about disability is based on themselves and their experiences, so their process of meaning‐making filters through these same layers.

Importantly, the biopsychosocial model is a research concept, though it is applied in rehabilitation care around measuring patients' functions and setting targets for peoples with disability. The collaborators' preference for this concept and relationship to it illustrates that complex research terms can offer helpful tools for those with relevant lived experiences to reflect on their own identity, in and beyond rehabilitation and care. It illustrates a blurrier relationship between the public and researchers—typically bifurcated as two discrete groups, yet this process has revealed that a concept like the biopsychosocial model is also relevant and serves compelling purposes of understanding and analysing experiences for someone with limited research experience who is often placed on the assumed ‘public’ branch of this bifurcation.

Considering the discussions around outputs and the relevance of research concepts to the public, some examples already exist regarding conceptual models of disability in more public spheres. Several examples of animations and videos explaining some of these conceptual models are available on YouTube,^52^, ^53^, ^54^ many of which are posted and created by disability‐related organisations like Scope. Notably, Scope even includes a video and extensive explanations of the social model on their website.^55^

Regarding outputs and applications of learnings and findings, public collaborators offered logical considerations about where and with whom to share research findings and learnings, such as disability charities, but they also offered compelling and unique options for applications, such as HR departments. The interest in HR departments especially, and beyond more anticipated contexts like disability charities, illustrates how widely applicable research on disability concepts can be to the broader world, especially through its focus on disability's conceptual complexity and impact on disabled peoples' lives.

## 5 STRENGTHS AND LIMITATIONS

This study's limitations included a relatively small group of public collaborators, though this was balanced and complemented by the strong and in‐depth degrees of co‐production, public involvement and relationship building with collaborators and co‐facilitators throughout the whole process of the activities.

This study's strengths include applying PPIE methods to a unique and complex research activity, typically not extended to members of the public with lived experiences, including representation of various disabilities, collaboratively planning, facilitating, analysing and writing up the activities and the prioritisation that authors gave to inclusivity throughout the entire study process.

## 6 CONCLUSION

In this PPIE activity, we aimed to conduct a set of PPIE consultations to gain some understanding of public perceptions of theoretical models of disability in survey questions. Throughout the process, all collaborators felt empowered, engaged and supported, which included unique activities on Explanation, Mapping, Reflection and Evaluation. We offer this approach as a model and a call for other researchers to give the public with lived experiences of disability more power and control over technical aspects of research typically reserved for researchers. Above all, our findings show that research concepts, when communicated and discussed in supportive and open environments, can help fulfil a needed role of supporting people think about their own disability, a key part of daily life that touches almost every aspect of living, though not necessarily and exclusively tied to a specific aspect like assessing functioning in care or rehab. More importantly, involving people with lived experience in the way that these research concepts are used and applied helps create understandings of disability and broader phenomena based on the people most intimately connected to and affected by it.

## AUTHOR CONTRIBUTIONS

**William Lammons**: conceptualisation; investigation; funding acquisition; writing—original draft; methodology; validation; writing—review and editing; software; formal analysis; project administration; data curation. **Lucky Nobility**: methodology; validation; writing—review and editing; software; formal analysis; conceptualisation; investigation; writing—original draft. **Sarah Markham**: conceptualisation; investigation; writing—original draft; methodology; validation; writing—review and editing; software; formal analysis. **Eirini‐Christina Saloniki**: conceptualisation; investigation; funding acquisition; writing—original draft; writing—review and editing; validation; methodology; software; formal analysis; data curation; supervision; project administration; resources.

## CONFLICT OF INTEREST STATEMENT

The authors declare no conflict of interest.

## ETHICS STATEMENT

Ethical approval for PPIE activities is not required, per the NIHR.11 As explained previously, public collaborators consented to participating in the activities, collaborating on the study and recording of the sessions.

## Supporting information

Supporting information.

## Data Availability

All relevant data are included in this manuscript. Additional anonymised transcript data were available upon reasonable request.
