# Supplementary material for: PPIE in a technical research study: Using public involvement to refine the concept and understanding and move towards a multidimensional concept of disability
Source: Health Expect. 2024 May 15;27(3):e14072. doi: 10.1111/hex.14072 (PMC11094671; doi:10.1111/hex.14072)
Supplement: Supplementary file 1 — Supporting information. [file HEX-27-e14072-s001.docx]

# Supplementary Material 1 – Topic Guides for Discussions

## Activity 3, Reflection

Which information would be useful for your groups?

Who and how do you think could benefit from this? Charities? Researchers? Future PPI activities?

What things could we share with other people? (We have a limited budget so what kind of outputs would be valuable?)

How else can we run this study into a larger sample?

## Activity 4, Evaluation and feedback

What did you like about the activities? What were the positives?

What could be better in the future?

How can we expand the study?

What were some of the challenges?

How did you feel about the recording?

What was the best moment in these sessions for you?

Would face to face be better?

# Supplementary Material 2 – Consolidated criteria for reporting qualitative research (COREQ)

| **Topic** | **Item No.** | **Guide Questions/Description** | **Reported on**  **Page No.** |
| --- | --- | --- | --- |
| **Domain 1: Research team**  **and reﬂexivity** | | | |
| *Personal characteristics* | | | |
| Interviewer/facilitator | 1 | Which author/s conducted the interview or focus group? | 3 |
| Credentials | 2 | What were the researcher’s credentials? E.g. PhD, MD | Authors page |
| Occupation | 3 | What was their occupation at the time of the study? | Authors page |
| Gender | 4 | Was the researcher male or female? | n/a |
| Experience and training | 5 | What experience or training did the researcher have? | Authors page |
| *Relationship with*  *participants* | | | |
| Relationship established | 6 | Was a relationship established prior to study commencement? | 3 |
| Participant knowledge of  the interviewer | 7 | What did the participants know about the researcher? e.g. personal  goals, reasons for doing the research |  |
|  |  |  | 3 |
|  |  |  |  |
| Interviewer characteristics | 8 | What characteristics were reported about the inter viewer/facilitator?  e.g. Bias, assumptions, reasons and interests in the research topic |  |
|  |  |  | 2, 3 |
|  |  |  |  |
| **Domain 2: Study design** | | | |
| *Theoretical framework* | | | |
| Methodological orientation and Theory | 9 | What methodological orientation was stated to underpin the study? e.g. grounded theory, discourse analysis, ethnography, phenomenology,  content analysis |  |
|  |  |  | 7, 8 |
|  |  |  |  |
| *Participant selection* | | | |
| Sampling | 10 | How were participants selected? e.g. purposive, convenience,  consecutive, snowball |  |
|  |  |  | 3 |
|  |  |  |  |
| Method of approach | 11 | How were participants approached? e.g. face-to-face, telephone, mail,  email |  |
|  |  |  | 3 |
|  |  |  |  |
| Sample size | 12 | How many participants were in the study? | 3, 8 |
| Non-participation | 13 | How many people refused to participate or dropped out? Reasons? | 3 |
| *Setting* | | | |
| Setting of data collection | 14 | Where was the data collected? e.g. home, clinic, workplace | 3-7 |
| Presence of non-  participants | 15 | Was anyone else present besides the participants and researchers? |  |
|  |  |  | 3-7 |
|  |  |  |  |
| Description of sample | 16 | What are the important characteristics of the sample? e.g. demographic  data, date |  |
|  |  |  | 3, 9 |
|  |  |  |  |
| *Data collection* | | | |
| Interview guide | 17 | Were questions, prompts, guides provided by the authors? Was it pilot  tested? | 4-7 |
|  |  |  |  |
| Repeat interviews | 18 | Were repeat inter views carried out? If yes, how many? | n/a |
| Audio/visual recording | 19 | Did the research use audio or visual recording to collect the data? | 4-7 |
| Field notes | 20 | Were ﬁeld notes made during and/or after the inter view or focus group? | 7, 8 |
| Duration | 21 | What was the duration of the inter views or focus group? | 4 |
| Data saturation | 22 | Was data saturation discussed? | 8 |
| Transcripts returned | 23 | Were transcripts returned to participants for comment and/or | n/a |

| **Topic** | **Item No.** | **Guide Questions/Description** | **Reported on**  **Page No.** |
| --- | --- | --- | --- |
|  |  | correction? |  |
| **Domain 3: analysis and**  **ﬁndings** | | | |
| *Data analysis* | | | |
| Number of data coders | 24 | How many data coders coded the data? | 8 |
| Description of the coding  tree | 25 | Did authors provide a description of the coding tree? |  |
|  |  |  | 8, n/a |
|  |  |  |  |
| Derivation of themes | 26 | Were themes identiﬁed in advance or derived from the data? | 7, 8 |
| Software | 27 | What software, if applicable, was used to manage the data? | 7, 8 |
| Participant checking | 28 | Did participants provide feedback on the ﬁndings? | n/a |
| *Reporting* | | | |
| Quotations presented | 29 | Were participant quotations presented to illustrate the themes/ﬁndings?  Was each quotation identiﬁed? e.g. participant number |  |
|  |  |  | 9-12 |
|  |  |  |  |
| Data and ﬁndings consistent | 30 | Was there consistency between the data presented and the ﬁndings? | 12-14 |
| Clarity of major themes | 31 | Were major themes clearly presented in the ﬁndings? | 9-12 |
| Clarity of minor themes | 32 | Is there a description of diverse cases or discussion of minor themes? | 9-12 |

Developed from: Tong A, Sainsbury P, Craig J. Consolidated criteria for reporting qualitative research (COREQ): a 32-item checklist for interviews and focus groups. *International Journal for Quality in Health Care*. 2007. Volume 19, Number 6: pp. 349 – 357

# Supplementary Material 3 - Guidance for Reporting Involvement of Patients and the Public, Version 2 (GRIPP2)

| **Appendix** **3** **– GRIPP2 long form** | | |
| --- | --- | --- |
| Section and topic | Item | Reported on page No |
| Section 1: Abstract of paper | | |
| 1a: Aim | Report the aim of the study | 2 |
| 1b: Methods | Describe the methods used by which patients  and the public were involved | 2 |
| 1c: Results | Report the impacts and outcomes of PPI in the  study | 2 |
| 1d:Conclusions | Summarise the main conclusions of the study | 2 |
| 1e: Keywords | Include PPI, “patient and public involvement,”  or alternative terms as keywords | 2 |
| Section 2: Background to paper | | |
| 2a: Definition | Report the definition of PPI used in the study  and how it links to comparable studies | 3, 4 |
| 2b: Theoretical  underpinnings | Report the theoretical rationale and any theoretical influences relating to PPI in the  study | 3, 4 |
| 2c: Concepts and theory  development | Report any conceptual models or influences  used in the study | 4-9 |
| Section 3: Aims of paper | | |
| 3: Aim | Report the aim of the study | 4 |
| Section 4: Methods of paper | | |

| 4a: Design | Provide a clear description of methods by which  patients and the public were involved | 4-9 |
| --- | --- | --- |
| 4b: People involved | Provide a description of patients, carers, and the public involved with the PPI activity in the  study | 4 |
| 4c: Stages of involvement | Report on how PPI is used at different stages of  the study | 4-9 |
| 4d: Level or nature of  involvement | Report the level or nature of PPI used at various  stages of the study | 4-9 |
| Section 5: Capture or measurement of PPI impact | | |
| 5a: Qualitative evidence of  impact | If applicable, report the methods used to qualitatively explore the impact of PPI in the  study | n/a |
| 5b: Quantitative evidence  of impact | If applicable, report the methods used to quantitatively measure or assess the impact of  PPI | n/a |
| 5c: Robustness of  measure | If applicable, report the rigour of the method  used to capture or measure the impact of PPI | 9 |
| Section 6: Economic assessment | | |
| 6: Economic assessment | If applicable, report the method used for an  economic assessment of PPI | n/a |
| Section 7: Study results | | |
| 7a: Outcomes of PPI | Report the results of PPI in the study, including | 9-13 |

|  | both positive and negative outcomes |  |
| --- | --- | --- |
| 7b: Impacts of PPI | Report the positive and negative impacts that PPI has had on the research, the individuals involved (including patients and researchers),  and wider impacts | 9-13 |
| 7c: Context of PPI | Report the influence of any contextual factors that enabled or hindered the process or impact  of PPI | 14-15 |
| 7d: Process of PPI | Report the influence of any process factors, that  enabled or hindered the impact of PPI | 14-15 |
| 7ei: Theory development | Report any conceptual or theoretical  development in PPI that have emerged | 12, 13 |
| 7eii: Theory development | Report evaluation of theoretical models, if any | n/a |
| 7f: Measurement | If applicable, report all aspects of instrument development and testing (eg, validity, reliability, feasibility, acceptability, responsiveness,  interpretability, appropriateness, precision) | n/a |
| 7g: Economic assessment | Report any information on the costs or benefit  of PPI | n/a |
| Section 8: Discussion and conclusions | | |
| 8a: Outcomes | Comment on how PPI influenced the study  overall. Describe positive and negative effects | 14, 15 |
| 8b: Impacts | Comment on the different impacts of PPI | 14, 15 |

|  | identified in this study and how they contribute  to new knowledge |  |
| --- | --- | --- |
| 8c: Definition | Comment on the definition of PPI used (reported in the Background section) and  whether or not you would suggest any changes | 3, 4, 14, 15 |
| 8d: Theoretical  underpinnings | Comment on any way your study adds to the  theoretical development of PPI | 14, 15 |
| 8e: Context | Comment on how context factors influenced PPI  in the study | 14, 15 |
| 8f: Process | Comment on how process factors influenced PPI  in the study | 14, 15 |
| 8g: Measurement and  capture of PPI impact | If applicable, comment on how well PPI impact  was evaluated or measured in the study | n/a |
| 8h: Economic assessment | If applicable, discuss any aspects of the economic cost or benefit of PPI, particularly any  suggestions for future economic modelling. | n/a |
| 8i: Reflections/critical  perspective | Comment critically on the study, reflecting on the things that went well and those that did not,  so that others can learn from this study | 14, 15 |
